# Classification of alkaloids according to the starting substances of their biosynthetic pathways using graph convolutional neural networks

**DOI:** 10.1186/s12859-019-2963-6

**Published:** 2019-07-09

**Authors:** Ryohei Eguchi, Naoaki Ono, Aki Hirai Morita, Tetsuo Katsuragi, Satoshi Nakamura, Ming Huang, Md. Altaf-Ul-Amin, Shigehiko Kanaya

**Affiliations:** 1 Division of Science and Technology, Graduate School of Science and Technology, Nara Institute of Science and Technology, Ikoma, Nara, 630-0192 Japan; 2 Data Science Center, Nara Institute of Science and Technology, Ikoma, Nara, 630-0192 Japan; 3 Department of Computer Science and Engineering, Toyohashi University of Technology, Hibarigaoka, Tempaku-cho, Toyohashi, Aichi, 441-8580 Japan

**Keywords:** Molecular graph convolutional neural networks, Alkaloids, Metabolic pathways, Deep learning

## Abstract

**Background:**

Alkaloids, a class of organic compounds that contain nitrogen bases, are mainly synthesized as secondary metabolites in plants and fungi, and they have a wide range of bioactivities. Although there are thousands of compounds in this class, few of their biosynthesis pathways are fully identified. In this study, we constructed a model to predict their precursors based on a novel kind of neural network called the molecular graph convolutional neural network. Molecular similarity is a crucial metric in the analysis of qualitative structure–activity relationships. However, it is sometimes difficult for current fingerprint representations to emphasize specific features for the target problems efficiently. It is advantageous to allow the model to select the appropriate features according to data-driven decisions for extracting more useful information, which influences a classification or regression problem substantially.

**Results:**

In this study, we applied a neural network architecture for undirected graph representation of molecules. By encoding a molecule as an abstract graph and applying "convolution" on the graph and training the weight of the neural network framework, the neural network can optimize feature selection for the training problem. By incorporating the effects from adjacent atoms recursively, graph convolutional neural networks can extract the features of latent atoms that represent chemical features of a molecule efficiently. In order to investigate alkaloid biosynthesis, we trained the network to distinguish the precursors of 566 alkaloids, which are almost all of the alkaloids whose biosynthesis pathways are known, and showed that the model could predict starting substances with an averaged accuracy of 97.5%.

**Conclusion:**

We have showed that our model can predict more accurately compared to the random forest and general neural network when the variables and fingerprints are not selected, while the performance is comparable when we carefully select 507 variables from 18000 dimensions of descriptors. The prediction of pathways contributes to understanding of alkaloid synthesis mechanisms and the application of graph based neural network models to similar problems in bioinformatics would therefore be beneficial. We applied our model to evaluate the precursors of biosynthesis of 12000 alkaloids found in various organisms and found power-low-like distribution.

## Background

The term “alkaloid” was introduced by German pharmacist Wilhelm Meissner and traditional definitions of alkaloids emphasized their bitter taste, basicity, plant origin, and physiological actions. The presence of at least one nitrogen atom is a general chemical feature of the alkaloids [1]. Alkaloids have extremely divergent chemical structures including heterocyclic ring systems and they encompass more than 20,000 different molecules in organisms [2]. To facilitate a systematic understanding of the alkaloids, the species–metabolite relation database (KNApSAcK Core DB [3]) has been established. To date, KNApSAcK Core DB includes 12,243 alkaloid compounds [4–6]. Alkaloids can be classified according to the starting substances of their biosynthetic pathways, such as the amino acids that provide nitrogen atoms and part of their skeleton including terpenoids and purines [7]. Thus, identification of starting substances that synthesize a variety of alkaloids is one of the most important keys for the classification of natural alkaloid compounds. Chemical structures of alkaloids are very diverse and the extraction of features of chemical compounds from molecular structures is crucial for the classification of alkaloid compounds. Although several chemical fingerprinting methods have been developed for prediction of the chemical and biological activities of alkaloids, the disadvantages of these methods lie in the fact that these kinds of fingerprints have some redundancy in their representation, and therefore do not perform well in analysis of complicated chemical ring systems [8–10]. For example, in the path-based fingerprint “FP2” implemented in Open Babel [11], chemical structures are represented by a bit string of length 1024 or longer, which represents all linear and ring substructures ranging from one to seven atoms, excluding the single-atom substructures of C and N. The circular fingerprint “ECFP” (extended-connectivity fingerprint) is a 1024-bit code mapped by a hashing procedure from circular neighboring atoms in a given diameter [12]. Moreover, there are projects to provide comprehensive sets of chemical descriptors, for example, PaDEL descriptor generator provides 1875 descriptors and and 12 types of fingerprints (total 16092 bits) [13]. However, those variables are not always important or relevant with the target features so that feature selection and optimization is indispensable. In the classification of alkaloids, these techniques to extract features from chemical structures were insufficient because of the diverged heterocyclic nitrogenous structures; i.e., 2546 types of ring skeleton were detected in 12,243 alkaloids accumulated in KNApSAcK Core DB [6]. Here, the ring skeleton means the ring system in a chemical compound detected in a simple graph representation of a chemical.

Thousands of physical and chemical parameters have been proposed to describe chemical features of organic compounds, and the evaluation of selections from those feature variables based on the optimized regression or on the classification for target variables is complex. In this study, we propose a classification system of alkaloids according to their starting substances based on a graph convolutional neural network (GCNN), which is a model that generalizes convolution operation for abstract graph structures, instead of the operations on 1D or 2D grids of variables that are commonly used in convolutional neural networks (CNN) [14, 15]. GCNN can be applied to arbitrary network structures, and molecular graph convolutional neural networks (MGCNN) are a classification and regression system that can extract molecular features from their structure [16–19]. This model focuses on the combination of atoms and their neighbors, and regards their molecular structures as a graph. Chemical descriptors for physicochemical features of compounds have long been discussed in research on chemoinformatics. Such descriptors are mainly used as inputs of machine learning or statistical analysis, in which various models and thousands of features including the number of bases and substructures, electric atmosphere, and so on have been proposed [20]. However, the significance of these features should depend on the specific problem and the selection of optimal features is required; otherwise, most of the variables would become a source of noise for statistical analysis.

The advantage of applying GCNN to the chemical structure is automatic optimization of the structural features; in other words, various combinations of local groups of atoms in some ranges can be considered through the weights of neural networks. In each convolution step, the weighted sum of feature vectors only in the adjacent atoms will be taken into account. By applying the convolution filters multiple times, we can gather information of neighboring atoms recursively, so an MGCNN can extract local molecular structures such as circular fingerprints. Moreover, during the training stages, the weights on the feature filters will be optimized for the target task. Therefore, we do not need to count unimportant or uncorrelated fingerprints and can focus on the features within appropriate ranges.

In this study, we applied the MGCNN model for classification of alkaloids, to understand their biosynthetic processes. Given that the biosynthesis pathways of alkaloid families as secondary metabolites in plants, microorganisms, and animals are so diverse and complex, it is worth computing to estimate “the starting substances” of each alkaloid from its molecular structures. By using alkaloids for which biosynthesis pathways are known as a training data set, the MGCNN model is trained to classify them into the categories defined by the starting compounds, e.g., amino acids, isopentenyl pyrophosphate, etc. Note that when an alkaloid is synthesized by combining several precursors, it will be classified into multiples categories. We further applied the trained model for the remaining alkaloids whose biosynthesis pathways are not clear, to predict the starting compounds of their synthesis.

## Methods

### Fingerprints

We verified the performance of our model with two descriptor sets using two machine learning models. The descriptors were Extended-Connectivity Fingerprint (ECFP) and PaDEL-Descriptor [13]. For ECFP, we composed 1024-bit fingerprint with diameter 2. For PaDEL descriptor, we generated 1D, 2D descriptors and all fingerprints obtaining 17968 variables in total. We first removed all non-informative variables, whose values are identical for all samples. Next, we computed the correlation matrix and constructed networks connecting highly correlated (*r*>0.6) variables. We found that the links of the correlated variables composed of 507 connected components. Then we randomly selected one variables from each connected component of the correlation network. We applied Random Forest (RF), Neural Networks (NN), and also kernel Support Vector Machine (SVM) by optimizing hyperparamters based on grid-search using these selected variables using “caret” packages in R software [21].

### Molecular graph convolution

**Fig. 1:**
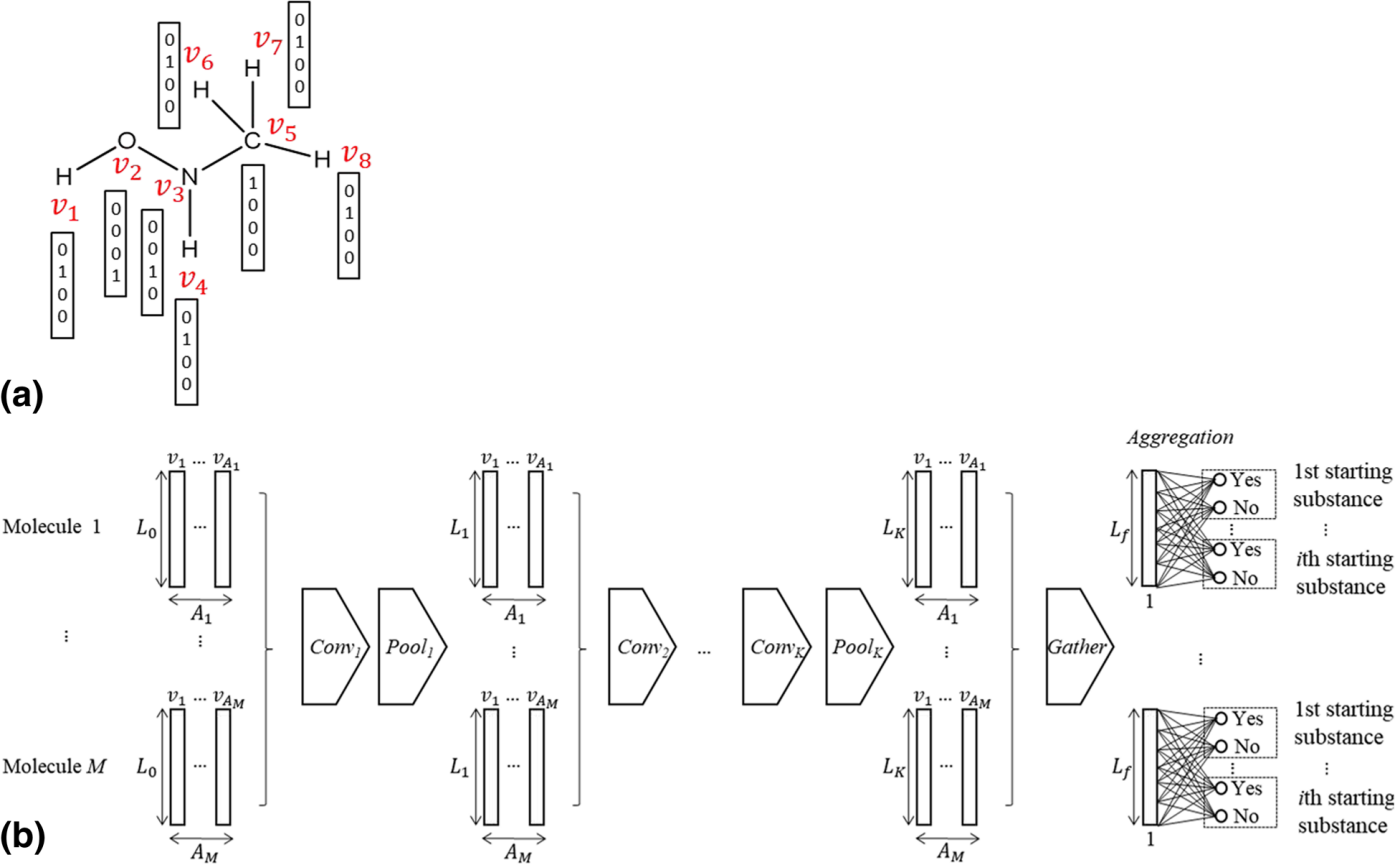
**a** Explanation of one-hot vectors for a molecule. **b** Schematic diagram of MGCNN (details are given in the text). In the case of the molecule shown in (**a**), the column number of input layer (*A*_*i*_) in (**b**) will be 8

Figure 1 shows a schematic diagram of MGCNN, which consists of convolution, pooling, and gathering. Convolution and pooling operations are repeated for three times to cover local molecular substructures. In MGCNN, molecular structures are described as abstract graphs, i.e., vertices as atoms and edges as chemical bonds, respectively.

As the initial input, atoms are represented by one-hot vectors that represent atom types. For example, if all molecules are composed of atoms {C,H,N,O}, one-hot vectors for the corresponding atoms can be represented by *C*=[1 0 0 0]^*T*^, *H*=[0 1 0 0]^*T*^, *N*=[0 0 1 0]^*T*^, and *O*=[0 0 0 1]^*T*^, respectively (Fig. 1a). Then, stages of convolution and pooling layers are applied to extract feature vectors (Fig. 1b). The feature vectors of all atoms are gathered in a single vector and applied for the classification of alkaloids according to their starting substances.

### Convolution and Pooling

**Fig. 2:**
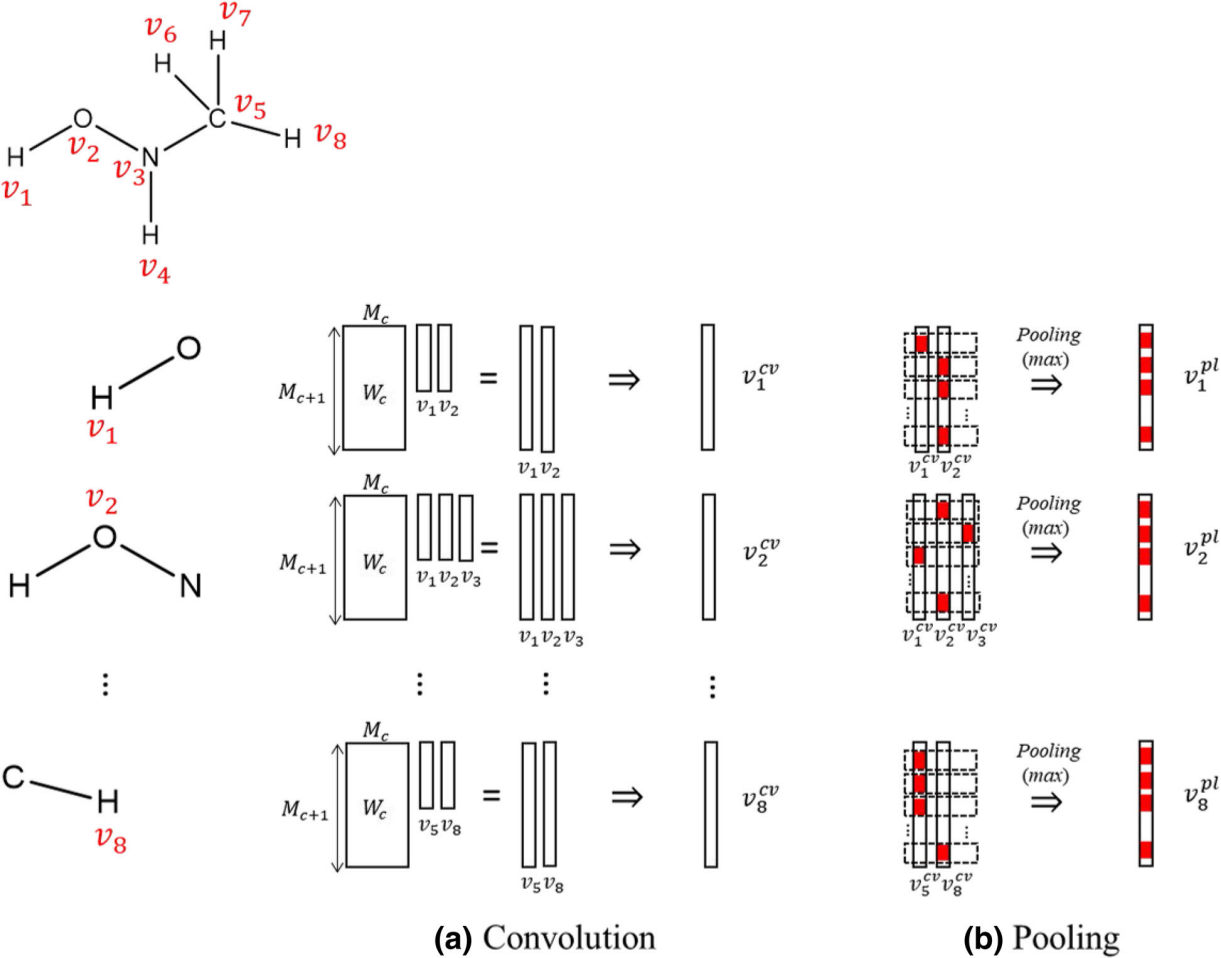
**a** Convolution and **b** pooling layers

As shown in Fig. 2, in MGCNN, convolution and pooling layers are coupled to gather information from neighboring atoms. A convolutional filter in MGCNN (Fig. 2b) is defined by Eq. (1):

1 $$ v_{i}^{c+1}=f_{ReLU}\left(\sum_{j \in {Adj(i)}} W_{c}(d) v_{j}^{c} \right),  $$

where $v_{j}^{c}$ is the vector of *i*th vertex as the input from the *c*th layer, *W*_*c*_(*d*) is the weight of the *c*th convolution layer, which depends on the distance *d* between the *i*th and *j*th vertices, *Adj*(*i*) gives a set of adjacent vertices of *i*th vertex (including the *i*th vertex itself), and *f*_*ReLU*_ is the activation function known as the rectified linear unit (ReLU) function [22]. Unlike convolution in regular grids, the number of adjacent vertices depends on the molecular structures. Thus, the output vector of the convolution layer ($v_{i}^{c+1}$) is determined by taking into consideration the relationships between neighboring atoms. In the pooling layers (Fig. 2b), updating of feature vectors for atoms is performed by comparing values $v_{j}^{c+1}$ for each row of the neighbors of the vertex *i*. In the present study, we chose the maximum values for each row called max pooling in Fig. 2b, where the red box represents the maximum value of each element. We evaluated several different numbers of convolution stages, i.e., pairs of convolution and pooling layers changing from one to six stages. The length of the feature vector in the last convolution layer is set to 128. Furthermore, dropout [23] of 80% is applied for the input layer, and 20% after each pooling layer to avoid overfitting.

### Gather and classification

A gather layer is applied after the series of convolution stages. In the gather layer, the final vector of the compound is represented as the sum of the feature vectors from all atoms. Then the molecular feature vector is passed as the input for the networks for classification. Note that some alkaloids are synthesized from combinations of several starting substances. Therefore, the output of the classification is represented as pairs of (*P*_*k*_ (positive) and *N*_*k*_ (negative)) nodes for each category *k* corresponding to the *k*th starting substance. Corresponding training labels are given by a binary vector $\mathbf {y}_{k} = (\hat {y}_{kp}, \hat {y}_{kn})$. In the output layer, the set of output vector {**y**_*k*_} is applied with a softmax function [24] and converted into a probability value independently for each category, respectively, so that one compound can be classified into multiple (or no) categories. The loss function $L(\{\mathbf {y}_{k}\}, \{\hat {\mathbf {y}}_{k}\})$ of the whole network is defined as the sum of cross entropy of predictions for all starting substances [25], as bellow,

2 $$ L(\{\mathbf{y}_{k}\}, \{\hat{\mathbf{y}}_{k}\}) = -\sum_{k=1}^{K} \left\{ \hat{y}_{kp}\log({y}_{kp})+\hat{y}_{kn}\log({y}_{kn}) \right\}.  $$

We trained the weights in the convolution layers by optimizing the weight parameters [26]. The goal of learning in the MGCNN model is to optimize the loss function *L* by updating the weights in the convolution layer [27, 28]. In the present study, the Adam (adaptive moment estimation) [29] method was used for updating because it works well in practice and compares favorably to other stochastic optimization methods. We evaluated the performance of the model by five fold cross-validation (CV5) and leave-one-out cross-validation (LOOCV). Since the loss function converged after around 100 epochs in almost all training data set, we fixed the number of epochs in every validation to 300.

### Data set

The training data used in this study are alkaloids for which chemical structures and secondary metabolic pathways are known. Secondary metabolic pathways of alkaloids were constructed based on the scientific literature and KEGG [30, 31], and are open to the public online at the KNApSAcK Database Portal as CobWeb Database ([32]). In this study, we used a total of 849 training samples corresponding to 566 alkaloids, which belong to 15 starting substances (Table 1); i.e., nine amino acids, L-alanine (abbreviated by L-Ala), L-arginine (L-Arg), L-aspartate (L-Asp), L-histidine (L-His), L-lysine (L-Lys), L-phenylalanine (L-Phe), L-proline (L-Pro), L-tryptophan (L-Trp), and L-tyrosine (L-Tyr); one aromatic acid, anthranilate; and four terpenoids, secologanin, isopentenyl diphosphate (IPP), geranylgeranyl diphosphate (GGPP), cholesterol; and the other, indole-3-glycerol phosphate (IGP). It should be noted that, in the training samples, 316 alkaloids are produced by single starting substances (ID = 1, 10, 12, 14, 15, 20, 24, 26, 28 in Table 1) and the remaining 533 training samples are produced by multiple starting substances.

**Table 1:** Data set used in this study

ID	Starting Substance	L-Ala	L-Arg	L-Asp	L-His	L-Lys	L-Phe	L-Pro	L-Trp	L-Tyr	Ant	Sec	IPP	GGPP	Cho	IGP
1	L-Ala	11														
2	L-Ala, L-Trp	2							2							
3	L-Ala, Anthranilate	1									1					
4	L-Ala, L-Pro, L-Trp, IPP	2						2	2				2			
5	L-Ala, L-Trp, Anthranilate	8							8		8					
6	L-Arg, L-Asp, Anthranilate		1	1							1					
7	L-Arg, L-Asp, L-Lys		1	1		1										
8	L-Arg, L-Asp, L-Phe, L-Pro		4	4			4	4								
9	L-Arg, L-Asp, L-Pro		7	7				7								
10	L-Arg, L-Pro		28					28								
11	L-Asp			12												
12	L-Asp, Anthranilate			1							1					
13	L-His				8											
14	L-His, L-Trp				11				11							
15	L-Lys					49										
16	L-Phe						5									
17	L-Phe, Anthranilate						6				6					
18	L-Phe, L-Tyr						7			7						
19	L-Pro, Anthranilate							4			4					
20	L-Pro, L-Trp							26	26							
21	L-Trp								53							
22	L-Trp, Anthranilate								11		11					
23	L-Trp, IPP								24				24			
24	L-Trp, Secologanin								56			56				
25	L-Tyr									129						
26	L-Tyr, Secologanin									27		27				
27	Anthranilate										30					
28	GGPP, IGP													25		25
29	Cholesterol														17	
Total	847	24	41	26	19	50	22	71	193	163	62	83	26	25	17	25

Anthranilate, secologanin, and cholesterol are abbreviated as Ant, Sec, and Cho, respectively

## Results

### Single classification in the MGCNN model

**Fig. 3:**
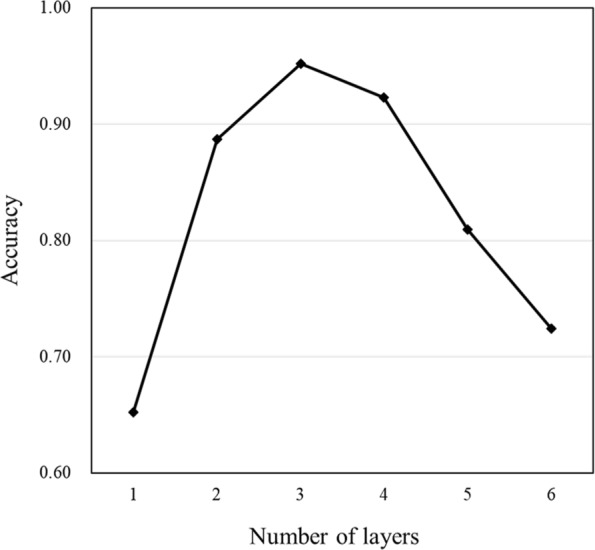
Accuracy for the number of layers

We evaluated the accuracy of the prediction of starting substances by changing the network size, i.e., the number of convolution stages, from one to six (Fig. 3). The best accuracy was obtained by the three-stage networks. Considering this result, we fixed the number of convolution stages to three in the following analysis.

**Fig. 4:**
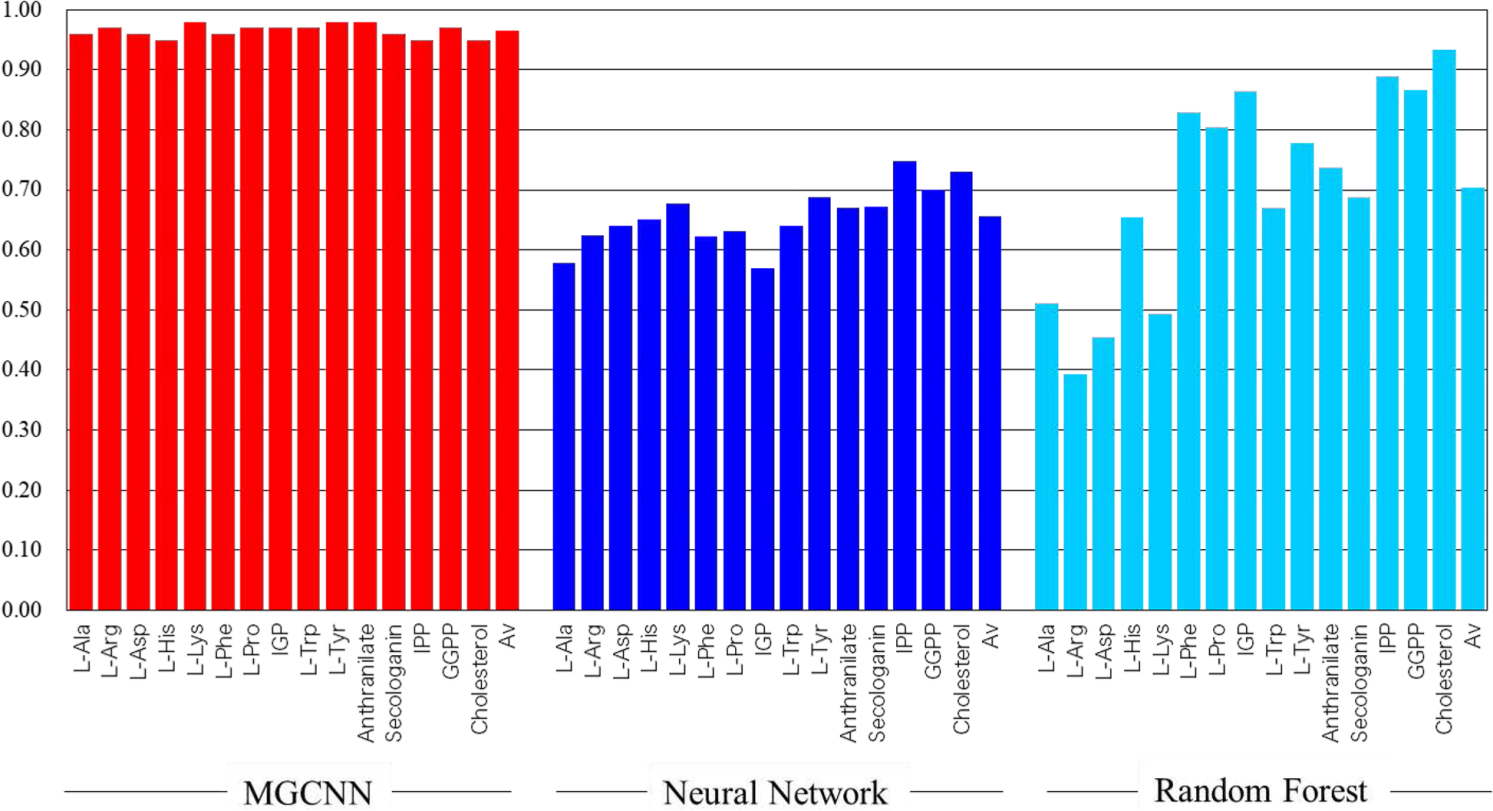
Accuracy for MGCNN, neural network, and random forest

To examine the effectiveness of MGCNN, we compared the prediction accuracy of MGCNN with a random forest [33] using a chemical fingerprint, namely 1024-bit ECFP (extended-connectivity fingerprint) [12], since a random forest is a commonly used method for classification and regression [34]. We also compared our method with a neural network with the same chemical fingerprint [35, 36] to evaluate the advantages of the graph representation. Figure 4 shows the accuracy of the classification for each of the 15 starting substances and their global average (*Av*) using the three methods evaluated by LOOCV. The global averages were 95.2% for MGCNN, 65.6% using the neural network model with ECFP, and 70.4% with the random forest. Notably, the performance of the random forest with ECFP varied widely among the starting substances, implying that the importance of the information depends greatly on the target problem. In contrast, MGCNN could classify alkaloids better compared with the random forest and the neural network with molecular fingerprint for all starting substances. We confirmed the prediction of MGCNN by CV5 and the accuracy for each starting substances were in the range 94.7*%* 99.6*%* and the average was 97.5%.

We also compared the performance of the network with using the selected PaDEL descriptors and fingerprints. Though the PaDEL descriptors and fingerprints composed of around eighteen thousands variables, most of them were non-informative for our alkaloid datasets, or, highly correlated with each other. We chose 507 variables by removing those non-informative variables beforehand (detail procedure is explained in “Fingerprints” section and applied RF, NN and SVM. The results showed very high accuracy (96.2%, 93.4%, and 96.5% respectively) but still significantly lower than that of MGCNN (*p*<0.001). This result implies that feature selection is quite effective for improvement of prediction accuracy of pathway classification and it is reasonable because the structures of molecular skeletons depend on mainly difference of biosynthesis processes and it can be described by choosing corresponding fingerprint variables.

### Multiclassification in the MGCNN model

The model was trained as a multilabel classifier; i.e., it was trained for each label independently. In the biosynthetic process of alkaloids, several compounds are biosynthesized from multiple starting substances; e.g., nicotine is synthesized from multiple starting substances, L-Asp and L-Arg. In practical applications using prediction of starting substances, it is important to evaluate the difference in the number of starting substances between training and predicted alkaloid compounds. Over 44% of the alkaloids were biosynthesized from multiple starting substances (average, 1.49), which is comparable with the results of the present model (average, 1.70). In fact, relationships between the predicted (*pr*) and original numbers (*no*) of starting substances can be regarded as *pr*=*no* with 95% confidence interval (the correlation coefficient *r*=0.97,−48.4<intercept<87.8,0.43<slope<1.21).

**Fig. 5:**
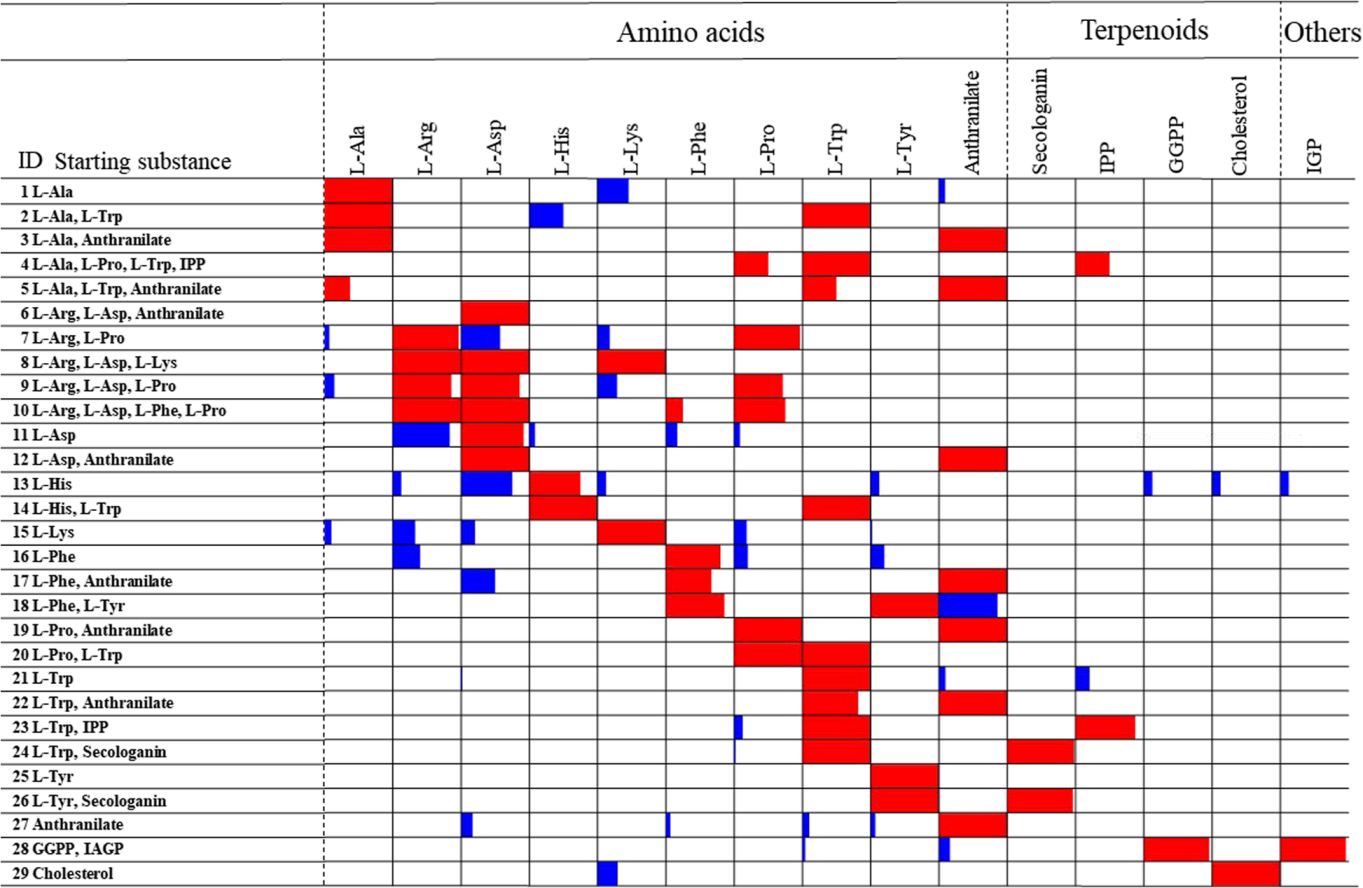
Classification of alkaloid compounds into 30 categories of starting substances. The width of the bar is set by 0 and 1. Classification rates are represented by red bars and misclassification rates by blue bars

Multilabeled classification by MGCNN was precise, and alkaloid compounds in most of the categories of starting substances (ID = 3–8, 14, 19, 20, 22, 24–26 in Fig. 5) were correctly classified. Here, the range of the histogram is set between 0 and 1, and classification rates are represented by red bars and misclassification rates by blue bars.

**Fig. 6:**
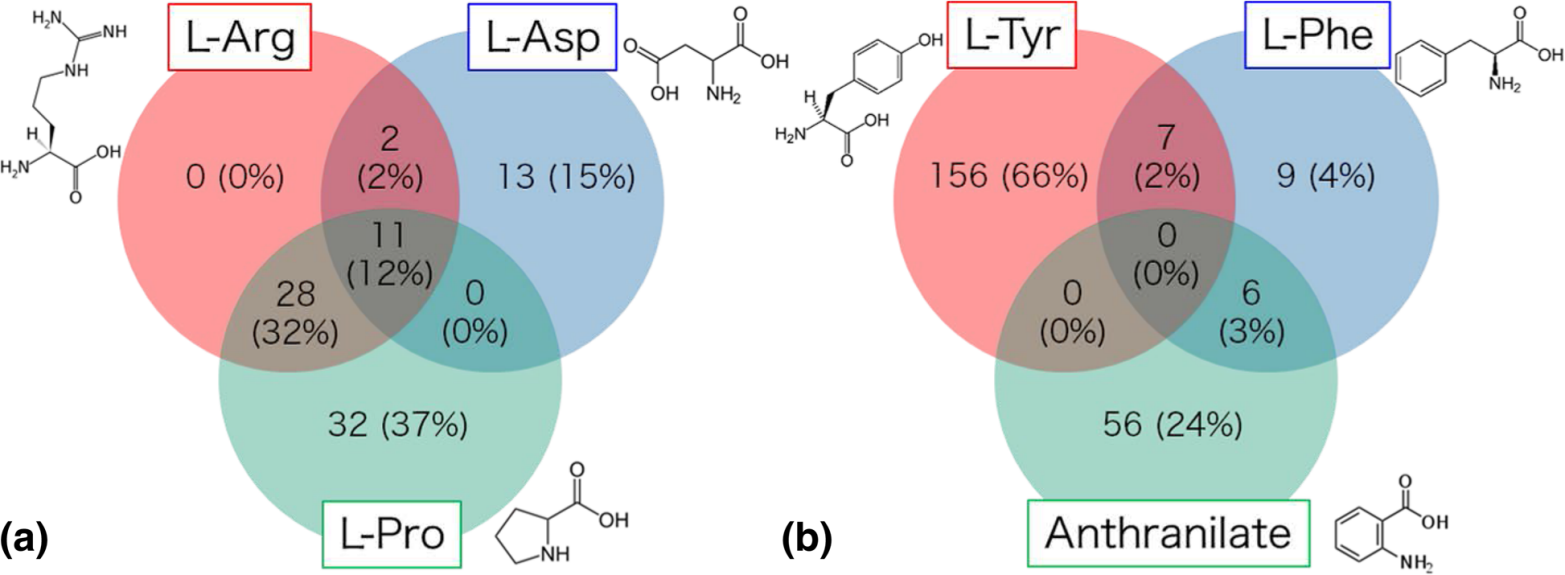
Examples of the number and percentage of compounds from multiple starting substances. **a** Combinations of L-Arg, L-Asp, and L-Pro. **b** Combinations of L-Tyr, L-Phe, Anthranilate

L-Arg and L-Pro are the starting substances for alkaloids of category 10, and L-Asp is the starting substance for alkaloids of category 11. In most cases, our approach correctly predicted starting substances for these two categories of alkaloids. However, in some cases, we observed the trend that L-Asp and L-Arg were predicted as starting substances of alkaloids of categories 10 and 11, respectively. It is well known that L-Pro, L-Asp, and L-Arg are highly associated in the secondary biosynthetic pathways; i.e., pyridine alkaloids [37], tropane alkaloids [38], and cocaine alkaloids [39] are biosynthesized from L-Pro, L-Asp, and L-Arg. The biosynthetic pathways from L-Pro, L-Asp, and L-Arg are displayed in alkaloid biosynthetic pathways in the KNApSAcK CobWeb. The numbers of alkaloids starting from L-Arg, L-Asp, and L-Pro and those from L-Tyr, L-Phe, and anthranilate in the training data are shown in Fig. 6. In total, 46% of alkaloids involving starting substances L-Arg, L-Asp, and L-Pro are synthesized from multiple substances (Fig. 6a).

In the case of category 18, most alkaloids were correctly assigned to L-Tyr and L-Phe as starting substances but tended to be misclassified as anthranilate. Otherwise, in the case of category 17, some alkaloids were correctly assigned to L-Phe and anthranilate, but some were wrongly assigned to L-Tyr. Three starting substances, L-Phe, L-Tyr, and anthranilate are commonly biosynthesized from chorismate [40], and those chemical structures are very similar to each other [41]. Only 3% of alkaloids were biosynthesized from a combination of those three starting substances (Fig. 6b) and a priority of classification of L-Tyr to L-Phe was observed in the MGCNN model because the chemical graph of L-Tyr includes that of L-Phe.

## Discussion

### Diversity of natural alkaloids based on starting substances predicted by the MGCNN model

**Fig. 7:**
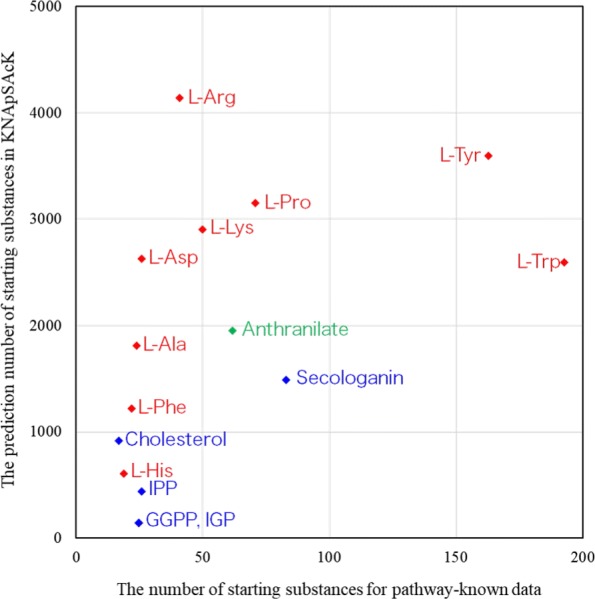
Relationship of the number of metabolites assigned to starting substances between pathway-known metabolites (training data) and metabolites in KNApSAcK Core DB. Amino acids, terpenoids, and others are represented in red, blue, and green, respectively

Estimation by MGCNN of the starting substances of alkaloid biosynthesis is a remarkable topic with respect to examining chemical diversity because, generally, although the chemical structures of alkaloids are known, their metabolic pathways are not. KNApSAcK Core DB [4, 5] has stored 116,315 metabolite–species pairs and 51,179 different metabolites. Of them, 12,460 metabolites belong to alkaloid compounds, which is comparable with the estimation of the number of different plant-produced alkaloids (approximately 12,000 alkaloids) [42]. An evaluation of the numbers of alkaloids linked to different starting substances leads to information on the origin of the creation and evolution of alkaloid diversity. To this end, we applied the MGCNN model to 12,460 compounds in the KNApSAcK DB. Figure 7 shows the number of metabolites in KNApSAcK DB (test data) associated with specific starting substances based on predicted results by MGCNN against the corresponding number calculated based on metabolites with known pathways (training data). A large number of alkaloids originating from starting substances L-Tyr and L-Trp are included in the training data, and a large number of alkaloids are also assigned to L-Tyr (3589 alkaloids) and L-Trp (2589 alkaloids) by the MGCNN model. Otherwise, a relatively small number of alkaloids are known to originate from the starting substances L-Arg, L-Pro, L-Lys, and L-Asp according to the training data, but a large number of alkaloids were predicted to be associated with starting substances L-Arg (4139 alkaloids), L-Pro (3145 alkaloids), L-Lys (2901 alkaloids), and L-Asp (2625 alkaloids). It should be emphasized that these six starting substances that have been assigned to most of the KNApSAcK DB metabolites fundamentally contribute to creating chemically diverged alkaloids. Other starting substances, four amino acids, L-Ala, L-Phe, L-His, anthranilate; and four terpenoids, GGPP, IPP, cholesterol, and secologanin, play auxiliary roles to create chemically diverged alkaloids.

**Fig. 8:**
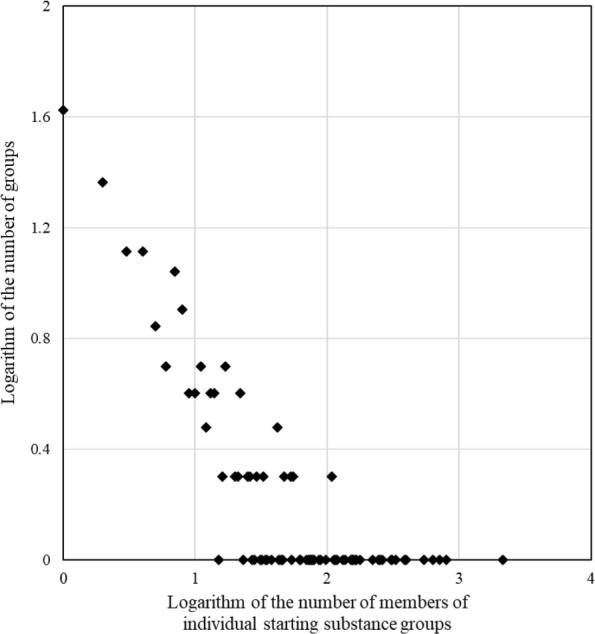
Relationships between the number of individual starting substance groups and the number of groups

In general, most alkaloids were predicted to be biosynthesized by multiple starting substances, which is consistent with the training data, in which 62% of alkaloids are biosynthesized by multiple starting substances. The combinations of predicted starting substances for the reported alkaloid data set can provide information about how to create chemical diversity. We evaluated the predicted starting substances of 12,460 alkaloids of KNApSAcK Core DB and observed 231 categories of combinations designated as starting groups. The MGCNN model did not assign any starting substances to just 263 alkaloids (2% of all alkaloids in the DB). Thus, the MGCNN model can provide important and useful information on starting substances. The relationship between the number of starting groups (y-axis) and the number of alkaloids in individual starting groups (x-axis) follows the power law (Fig. 8; *r*=−0.80).

**Fig. 9:**
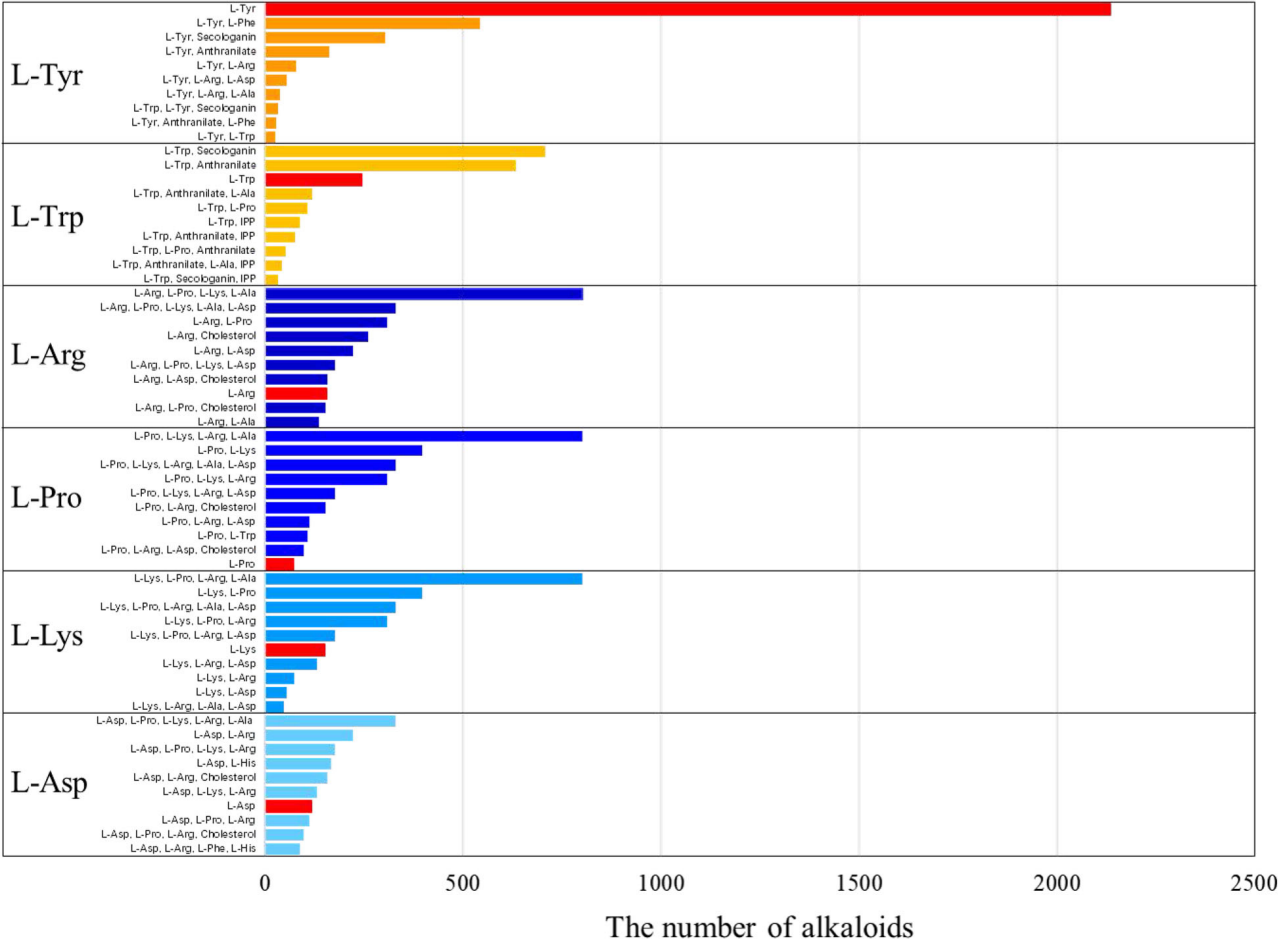
The 10 best combinations of the six major starting substances. The numbers of alkaloids with single starting substances are indicated as red bars

Figure 9 shows the 10 highest-frequency starting groups (combinations of starting substances) associated with each of the six major starting substances. Generally, L-Tyr is the starting substance to produce benzylisoquinoline alkaloids [42], spiroalkaloid alkaloids [43], catechol amines [44], and betalains [45]. Approximately 2500 elucidated chemical structures of benzylisoquinoline alkaloids have been reported and are known to have potent pharmacological properties [42, 46]. L-Tyr and anthranilate are associated with the tetrahydroisoquinoline monoterpene skeleton in alkaloids, including ipecac alkaloids [47]. The number of alkaloids biosynthesized by only L-Tyr as a starting substance is the largest (2135 alkaloids) (Fig. 9) and the number of alkaloids originating from a combination of L-Tyr and anthranilate ranked third (634 alkaloids). Thus, a large number of alkaloids are expected to be produced by L-Tyr and by a combination of L-Tyr and other chemical substances.

Nonribosomal peptide synthesis (NRPS) is a key mechanism responsible for the biosynthesis of diverged alkaloids in bacteria and fungi [48, 49]. The pairs of L-Trp and anthranilate, and of L-Trp and L-Pro are the starting substances in alkaloids that are produced by NRPS associated with fungal indole alkaloids [50]. L-Trp and secologanin are starting substances for producing monoterpenoid indole alkaloids, of which approximately 2000 compounds are widely used in medicine [42]. Among L-Trp-related groups (Fig. 9, L-Trp), the combination of L-Trp and secologanin produces the largest number of alkaloids (707 alkaloids), which is consistent with the diversity of monoterpenoid indole alkaloids [42]. The pairs of L-Trp and anthranilate, and of L-Trp and L-Pro also lead to diverged alkaloids (634 and 108 alkaloids, respectively) as reported in KNApSAcK Core DB. Only 244 alkaloids that are mainly associated with *beta*-carboline alkaloids [51] were classified to have a single L-Trp molecule as a starting substance. In the case of L-Trp, combinations of multiple starting substances tend to contribute to diverged alkaloid production, whereas in the case of L-Trp, it tends to serve as a starting substance by itself. Combinations of the three starting substances, L-Asp, L-Pro, and L-Arg, enable the biosynthesis of a very diverged array of alkaloids such as pyrrolizidine alkaloids [52], pyridine alkaloids, tropane alkaloids [53], and loline alkaloids [54], and combinations of these three starting substances and cholesterol also contribute to steroidal alkaloids. L-Lys combined with other amino acids including L-Ala, L-Arg, and L-Pro as starting substances biosynthesize diverged alkaloids. Furthermore, L-Lys alone is the starting substance to produce diverged alkaloids including quinolizidine, indolizidine, lycopodium, and piperidine alkaloids [55].

From the results obtained by MGCNN, we could evaluate and better understand the chemical diversity of alkaloid synthesis according to starting substances based on natural products and the species–metabolite relation database KNApSAcK.

### Comparison between MGCNN and fingerprint-based methods

According to the comparison shown in Fig. 4, classification results by the random forest was more accurate for larger molecules, e.g., cholesterol, while the neural network outperformed for smaller compounds such as amino acids. This implies that fingerprints provide information for larger molecules, but neural network can optimize weights to evaluate features even for smaller molecules. In many cases, the selection of relevant features and optimization of weight can greatly improve the performance of machine learning based on molecular fingerprints [56, 57]. Moreover, it has been shown that CNN on graphs can be trained to activate important fragments corresponding to different tasks such as solubility and toxicity prediction [58].

Kearnes and others have also compared machine learning and GCNN models [59] using public datasets such as PubChem BioAssay, Tox21 Challenge, and so on. The authors demonstrated that GCNN is less sensitive for the model parameters compared with fingerprint-based methods. Flexibility and adaptability are general advantages of the GCNN-based model. By changing the number of convolution layers, almost all possible features of local molecular structure can be extracted by using GCNN, and adjustable weights on those feature variables through the neural network allow the data-driven optimization of features depending on various target tasks. Although the present model only considers topological connections between atoms, further development of GCNN to take into account detailed 3D molecular structures will provide more quantitative prediction of molecular features.

## Conclusion

We have developed and applied the MGCNN model for the classification and prediction of the starting substances used in alkaloid biosynthesis. The model could predict starting substances of their pathways with an averaged accuracy of 97%; whereas the averaged accuracy of random forest and neural networks were 70% and 66%, respectively. On the other hand, when we selected informative variables from thousands of descriptors and fingerprints, the accuracy of Random Forest and simple Neural Networks showed more comparable accuracy. The results show that the model can classify individual alkaloids into the starting substance groups very accurately, even though it is a multilabeled classification problem that is generally more difficult than single-labeled classification. In the MGCNN, although we considered only abstract topological binding between atoms, the information of the neighboring atoms could be accumulated through feature extraction using stacked multiple convolution layers and the coefficient of the convolution filters could optimize the weights regarding which atoms should be focused on in each filter. By gathering information from each filter, the classification network could optimize the weights to learn the relationship between the extracted features and the chemical properties of the given molecules.

## Data Availability

All data analyzed in this study are available at http://kanaya.naist.jp/KNApSAcK_Family/. The python code for the MGCNN is available at https://github.com/naono-git/mgcnn_alkaloid.

## Abbreviations

Adam: Adaptive moment estimation
CNN: Convolutional neural networks
ECFP: Extended-connectivity fingerprint
GCNN: Graph convolutional neural network
LOOCV: Leave-one-out cross-validation
MGCNN: Molecular graph convolutional neural networks
NRPS: Nonribosomal peptide synthesis
ReLU: Rectified linear unit
